# Hh signaling in regeneration of the ischemic heart

**DOI:** 10.1007/s00018-017-2534-9

**Published:** 2017-05-18

**Authors:** Marina Dunaeva, Johannes Waltenberger

**Affiliations:** 1 Department of Biomolecular Chemistry, Institute for Molecules and Materials and Radboud Institute for Molecular Life Sciences, Radboud University Nijmegen, Nijmegen, The Netherlands; 2 Chair of Internal Medicine, Cardiology and Vascular Medicine, Division of Cardiology, Department of Cardiovascular Medicine, University Hospital Münster, Albert-Schweitzer, Campus 1, Building A1, 48149 Münster, Germany; 3 Cells-in-Motion Cluster of Excellence (EXC 1003—CiM), University of Münster, Münster, Germany

**Keywords:** Hedgehog, Heart regeneration, Cardiomyocytes, Neovascularization

## Abstract

Myocardial infarction (MI) is caused by the occlusion of a coronary artery due to underlying atherosclerosis complicated by localized thrombosis. The blockage of blood flow leads to cardiomyocyte (CM) death in the infarcted area. Adult mammalian cardiomyocytes have little capacity to proliferate in response to injury; however, some pathways active during embryogenesis and silent during adult life are recruited in response to tissue injury. One such example is hedgehog (Hh) signaling. Hh is involved in the embryonic development of the heart and coronary vascular system. Pathological conditions including ischemia activate Hh signaling in adult tissues. This review highlights the involvement of Hh signaling in ischemic tissue regeneration with a particular emphasis on heart regeneration and discusses its potential role as a therapeutic agent.

## Introduction

Myocardial infarction (MI) is caused by the occlusion of a coronary artery due to atherothrombosis. Blood flow to the heart muscle is blocked resulting in cardiomyocyte (CM) death in the infarcted area. As a consequence, progressive and negative left ventricular remodeling (LV) and scar tissue formation take place [1]. After MI many CMs are lost and replaced by activated fibroblasts that cannot contribute to the contractile activity of the myocardium. Thus, the ischemic myocardium requires regeneration of damaged tissues such as cardiac muscle as well as heart vasculature. In general, the human body has mechanisms to partially compensate these losses but they are insufficient to restore heart function. Adult mammalian heart CMs have little capacity to proliferate in response to injury. The majority of cardiomyocytes stops proliferation shortly after birth [2], although continuous cardiomyocyte turnover has been detected in adult mouse and human hearts [3, 4]. Enhancement of this endogenous capability to regenerate can be considered as a strategy to develop cardiac regeneration therapies.

It has become evident that some pathways active during embryogenesis and silent during adult life are recruited in response to tissue injury and regeneration. Among them is hedgehog (Hh) signaling. The process of regeneration includes such events as cellular proliferation, differentiation and dedifferentiation. Hh signaling controls both cell proliferation and differentiation and it is involved in cell cycle regulation. Hh is implicated in the regulation of embryonic heart and coronary vascular system development [5–7]. Hh signaling components are also present in adult cardiovascular tissues, although its activity is at a very low level. Pathological conditions such as ischemia reactivate Hh signaling. This review summarizes and discusses the data which support the role of Hh signaling in myocardial regeneration and blood vessel repair.

### Hedgehog signaling

Sonic hedgehog (Shh), Indian hedgehog (Ihh), and Desert hedgehog (Dhh) are secreted signaling proteins, which act as morphogens during embryonic development. The Hh signaling pathway is initiated by the binding of Hh proteins to Patched1 (Ptc1) or Patched2 (Ptc2) receptors. In the absence of Hh proteins, the 12-transmembrane receptor Ptc1 inhibits activity of smoothened (Smo), a 7-transmembrane protein that regulates activation of the Glioma-associated oncogene homologue (Gli) family of transcription factors; Gli1, Gli2, and Gli3 [8]. In the presence of Hh proteins, Ptc1 is internalized and Smo is released from repression. This step leads to the dissociation of a cytosolic complex with Glis and their subsequent translocation into nucleus to transduce Hh signaling. Gli1 itself, Ptc1 and Ptc2 are Gli-target genes and upregulation of their expression can serve as a marker for Hh signaling activation. Moreover, upregulation of Ptc1 and Ptc2 in response to Hh signaling creates a negative feedback loop to regulate Hh protein distribution and the level of Hh pathway activation. The activation of the pathway via Gli-dependent transcription is considered as a “canonical” response to Hh. Hh proteins can also stimulate Gli-independent pathways, which involve either Ptc1 activity without its inhibitory activity on Smo, or Smo activity without Gli regulation (noncanonical Hh signaling) [9].

## Hh in heart regeneration of lower vertebrates

### Zebrafish

As indicated above, the signaling pathways that regulate cardiac development are often reactivated during heart repair in response to injury. Several studies on zebrafish embryos implicated Hh signaling in cardiac development. Genetic or pharmacological inhibition or activation of Hh signaling in zebrafish embryos revealed that Hh signaling is involved in cardiomyocyte formation via specification of myocardial progenitors during gastrulation and early somitogenesis stages [10]. Furthermore, monitoring of cardiomyocyte proliferation in live zebrafish embryo using the *FUCCI* technology demonstrated the role of Hh signaling also in cardiac proliferation. The treatment of embryos with either Hh signaling Smoothened agonist (SAG) or antagonist cyclopamine (CyA) increased or reduced the number of proliferating cardiomyocyte, respectively [11]. These results clearly indicate that Hh signaling is crucial for cardiomyocyte formation and proliferation in the zebrafish embryo.

Zebrafish models have also been used to study the cardiac recovery in response to injury due to the ability to effectively regenerate their heart. To check whether heart injury activated Hh signaling during regeneration, Choi et al. [11] generated two transgenic reporter zebrafish lines in which enhanced green fluorescent protein (EGFP) expression was under the control of either Shh or Ptc2 promoter. Analysis of expression of Hh signaling components after partial ventricular resection of adult zebrafish heart has shown the upregulation of Shh in epicardial cells within the injury. Upregulation of *ptch2* in cardiomyocytes in the area of regeneration was a marker of Hh signaling activation. In addition, similar to the zebrafish embryo, treatment of animals with CyA for 6 days after ventricle resection or diffuse genetic ablation of cardiomyocytes displayed a decrease of cardiomyocyte proliferation.

A recent study on genetic depletion of the epicardium in adult zebrafish identified Hh signaling as a mediator of epicardial regeneration [12]. The epicardium depletion resulted in delay of the whole repair process. The heart regeneration after myocardial loss was completed only after the epicardium recovery which was dependent on Shh. Treatment with Shh enhanced epicardial response to injury, whereas inhibition of Hh signaling with CyA blocked regeneration of cardiac explants ex vivo through reduced epicardial cell proliferation promoted by endogenous Hh signaling.

### Newt

Likewise, adult newts have the ability to regenerate heart [13] as well as spinal cord and neuronal tissues [14], retina and lens [15], and limbs [16]. Involvement of Hh signaling in regeneration of lens, limb bud, tail, and heart of embryos and adult newts has also been investigated [17–20]. In general, injury resulted in upregulation of Shh and Ptc1 expression in regenerating tissues. In case of heart, the resection study on adult newt heart demonstrated upregulation of Shh and Ptc1 proteins in epicardial and myocardial cells [20]. Interference with Hh signaling led to repression of regeneration process, including cell proliferation.

Taken together, upregulation of Hh signaling as a response to heart injury is common for zebrafish and newt. It seems likely that Shh activated in epicardium is responsible for increased proliferation of cardiomyocytes via Ptc receptors.

## Hh in heart regeneration of mammals

### Fetal and neonatal rodents

Similar to zebrafish and amphibians, fetal and neonatal mice show a robust capacity for cardiac regeneration after injury [21, 22]. However, the neonatal mouse heart retains regenerative potential for only 7 days after birth [22].

Nothing is known about the involvement of Hh signaling in regeneration of fetal or neonatal rodent hearts. However, intact Hh signaling has been demonstrated in neonatal ventricular myocytes isolated from 1 to 3 days rat pups (NRVM). Incubation of NRVMs with recombinant Shh resulted in upregulation of *Gli1* and *Ptc1* genes and treatment with CyA abolished this response suggesting that these cells are Hh responsive [23]. Responsiveness of neonatal rat primary cardiac cells to Shh protein has also been observed by other groups [24, 25]. Pretreatment of these cells with either free recombinant Shh or incorporated in a coacervate, a delivery system comprised of heparin and a synthetic polycation, reduced apoptosis levels compared to the H_2_O_2_ treated control group. Together, these results indicate Hh signaling is functional in neonatal murine cardiomyocytes and might participate in cardiac regeneration in response to heart injury.

### Adult rodents

Hh signaling has also been implicated in adult heart homeostasis and in response to heart injury. In rodent adult heart expression of Hh signaling components, especially Ptc1, was detected in several resident cell populations: perivascular interstitial fibroblasts [26], myocardial fibroblasts and cardiomyocytes [24, 27] and endothelial, medial and adventitial cells of cardiovascular tissue vasculature [28]. Thus, these cells can be targets of Hh signaling.

The direct role of Hh signaling in homeostasis of adult heart is rather controversial. Inactivation of Hh signaling via Smo deletion in cardiomyocytes (Smo^mer^) or in cardiomyocytes and vascular smooth muscle cells (Smo^mer,sm22^) in mouse heart caused cardiomegaly, ventricular dilation, impaired systolic function and fibrotic replacement of myocardial tissue. Acute inhibition of myocardial Hh signaling led to heart failure and subsequent lethality due to coronary vascular disruption and tissue hypoxia suggesting that Hh signaling is critical for adult heart maintenance [26]. Another study reported that, although both Ptc1 and Smo were expressed in adult cardiomyocytes, inhibition of endogenous Hh signaling in rat adult hearts with CyA did not have any effect on electrocardiogram (ECG) properties, indicating no presence of active Hh signaling in the heart [29]. This apparent contradiction between the two studies may be explained by the fact that the elapsed time between treatment and examination of the animals differed. The animals in the latter study were treated with CyA for only 6 h before ECG analysis was performed. In comparison, 5 days passed between the beginning of the treatment to ablate Smo and subsequent analysis in the former study. Thus, the question whether active Hh signaling is present in adult heart is still open.

The presence of Hh signaling in adult heart and its involvement in homeostasis also prompted studies on the role of Hh signaling in response to cardiac ischemia. Several groups used a model of acute MI induced by surgical ligation of the left anterior descending artery (LAD). Myocardial ischemia reactivated Hh signaling as Shh and Ptc1 expressions were upregulated in the adult mouse heart [24, 27, 30]. Furthermore, intramyocardial injection of human Shh plasmid (phShh) immediately after LAD ligation significantly reduced the infarct and fibrosis areas, increased capillary density and α smooth muscle actin (αSMA) positive cell area in the ischemic zone and improved left ventricular function in rat hearts [24]. Likewise, intravenous injection of the recombinant Shh homolog ligand (N-Shh) led to reduction of the infarct area and subsequent arrhythmias [29]. In addition, both endogenous and exogenous Shh prevented cardiac apoptosis induced by myocardial ischemia [24, 30]. Inhibition of endogenous Hh with either neutralizing anti-mouse Shh antibody [25] or Hh signaling antagonist SANT-1 [27] after MI resulted in a significant increase of the infarct area, border zone tissue hypoxia, and a reduction in border zone coronary vessel density. Studies on mouse hindlimb ischemia provided similar results; activation of endogenous Hh signaling or administration of recombinant Shh or cells modified to express Shh stimulated neovascularization in different tissues (Table 1). Taken together, these results clearly show a protective role of Hh signaling in response to cardiac ischemia. Strikingly, Bijlsma et al. [30] have shown that inhibition of endogenous Hh signaling with CyA had a beneficial effect on ischemic myocardium in mice; it restored ventricular function and decreased fibrosis but it did not significantly affect vascularization. The reason for this discrepancy is unclear, the experimental settings were different and their direct comparison is rather difficult. The authors assumed that the role of Hh signaling might change during different phases of myocardial ischemia. However, studies on Hh function during different stages after infarction are needed to confirm this assumption.

**Table 1:** Effects of endogenous and exogenous Hh signaling on muscle regeneration

	Hedgehog signaling members	Effect	References
Mouse ischemic hindlimb model (NLS-*Ptch*-*lacZ* mice; surgically induced)	Endogenous Hh signaling activation	1. Upregulation of Shh mRNA in ischemic skeletal muscles particularly in the interstitial regions and Shh and Ptc1 proteins in interstitial mesenchymal fibroblasts 2. VEGF expression upregulation in interstitial mesenchymal fibroblasts 3. Inhibition of Hh signaling with Hh-blocking antibody (5E1) decreased blood flow and capillary density	Pola et al. [52]
Two mouse injury models: (1) mechanical crush; (2) cardiotoxin injection of the tibialis anterior (TA) muscle	Endogenous Hh signaling activation	1. In both models, Shh and Ptc1 mRNAs were upregulated. Shh-positive signal was detected in skeletal muscle fibers surrounding the injured area 2. Muscle satellite cells directly respond to Shh in the setting of muscle injury in vivo; Ptc1 is upregulated 3. Hh signaling inhibition results in impaired production of angiogenic and myogenic secreted factors, decreased upregulation of the Myf5 and MyoD, impairment of the angiogenic response to injury, reduction of the number of activated satellite cells at the damage site, increased fibrosis	Straface et al. [53]
Mouse ischemic hindlimb model (resection of the left femoral artery)	Endogenous Hh signaling activation and exogenous recombinant Shh administration	1. Gli2 and Gli3 mRNA were overexpressed in the ischemic tissue including myocytes and endothelial cells 2. Overexpression of Gli2 and Gli3 in vitro promotes myoblast survival and proliferation and EC survival and migration as well as induce expression of MMP-9 and osteopontin 3. Administration of the tibialis anterior muscles with adenovirus encoding Gli2 and Gli3 led to higher proliferation in the regenerating muscle	Renault et al. [54]
Mouse ischemic hindlimb model (Gli3 ± mice; resection of the left femoral artery)	Endogenous Hh signaling suppression	1. Gli3^+/–^ mice exhibit reduced capillary density 2. Gli3 overexpression in vitro led to increased Akt phosphorylation, activation of the ERK1/2 and increased c-Fos expression	Renault et al. [55]
Mouse ischemic hindlimb model (NLS-*Ptch*-*lacZ* mice, surgically induced)	Intravascular injection of exogenous recombinant Shh	1. The percentage of auto-amputated limbs and foot/leg necrosis significantly decreased 2. A progressive increase in the blood flow; increased numbers of capillaries, a substantial increase in vessel diameter 3. Shh upregulated Ptc1, VEGF and Ang2 in cultured fibroblasts	Pola et al.[28]
Mouse ischemic hindlimb model (1 year old mice)	Injection of a plasmid containing the amino-terminal domain coding the human Shh (phShh) in 5 separate sites of the hindlimb	1. Ischemia led to Gli1 upregulation in young but not middle-aged mice 2. Ptc1 is robustly expressed in large areas of phShh-treated middle-aged muscles, particularly in interstitial fibroblasts 3. phShh treatment induced complete recovery of blood flow in ischemic hindlimbs of middle-aged mice. Capillary and arteriole density was significantly increased compared with control 4. phShh treatment resulted in significant increase of the number of circulating bone marrow derived CD45^−^/Sca-1^+^/Flk-1^+^ cells 5. phShh treatment resulted in upregulation of VEGF, Ang-1 and SDF-1 in hindlimb muscles	Palladino et al. [56]
Mouse ischemic hindlimb model (18 month mice)	Combined phShh and endothelial progenitor cells (EPC) therapy (intramuscular injection)	1. Combined therapy resulted in a significant increase in capillary density compared to phShh gene transfer or EPC administration 2. phShh therapy increased the incorporation of transplanted EPCs into site of ischemia and reduced their apoptosis	Palladino et al. [57]
Mouse ischemic hindlimb model (age 8–10 week)	Shh-treated human peripheral blood derived CD34^+^ locally injected to lower limb muscles	1. Significant increase of blood perfusion ratio of ischemic/non-ischemic hindlimbs in mice treated with Shh-CD34^+^ cells 2. The treatment with SHh-CD34^+^ cells significantly increased capillary density compared with the control groups 3. Shh-CD34^+^ cells showed better incorporation into vascular structures in the ischemic limb muscles 4. Transplanted Shh-CD34^+^ cells expressed VEGF-A, while non-treated cells show no VEGF-A expression	Kanaya et al. [58]
Mouse ischemic hindlimb model (12-week-old Smo ± mice; resection of the left femoral artery)	Endogenous Hh signaling suppression	1. Ischemia-induced myogenesis and skeletal muscle repair were delayed in Smo ± mice compared with wild type mice.	Renault et al. [59]
Mouse ischemic hindlimb model (streptozotocin induced type 1 diabetic mice)	SAG treatment	1. SAG treatment increased capillary density and blood perfusion in the ischemic hindlimb of diabetic mice 2. SAG significantly increased the activity of AKT in EPCs	Qin et al. [60]

### Cell-based therapy

The results described above demonstrate that Hh signaling has a potential to preserve cardiac function and to influence cardiac recovery in the context of myocardial ischemia. To activate Hh pathway in ischemic heart, several cell-based therapeutic strategies have been applied. CD34^+^ cells are hematopoietic cells that have been previously used for stem cell therapy to preserve the functions of ischemic myocardium or to treat refractory angina [31]. When CD34^+^ cells, genetically modified to express Shh (CD34^Shh^), were injected into the border zone of mice with acute MI, they induced a robust increase in border zone capillary density accompanied by reduced infarct size in comparison with unmodified CD34^+^ cells or cells transfected with the empty vector [32]. Mesenchymal stem cells (MSCs) are potentially more pro-angiogenic and proarteriogenic than other types of stem cells [33]. Similar to CD34^Shh^ cells, injection of MSCs engineered to overexpress Shh (MSC^Shh^) in infarcted rat hearts resulted in increased capillary development and density within the infarct border zone, accompanied by reduced infarct sizes. Thus, Shh improved functional preservation of cardiac tissue compared with control cells [34]. Moreover, a high-number of mature blood vessels (smooth muscle actin^+^ cells) were observed in mice treated with MSC^Shh^. Furthermore, genetic modification of MSCs with Shh improved their survival and angiogenic potential in the ischemic heart via upregulation of VEGF, Ang-1, iNOS, netrin-1, hepatocyte growth factor (HGF), stromal cell-derived factor 1a (SDF-1a) and insulin-like growth factor-1 (IGF-1).

Recent studies on rodent models of acute and chronic MI reported that the effectiveness of pro-angiogenic gene therapy could be improved by its combination with progenitor cell mobilization. Indeed, Roncalli et al. [35] demonstrated that Shh gene therapy in combination with ADM3100-stimulated progenitor cell mobilization (ADM3100 is a pharmacological agent that mobilizes progenitor cells) reduced cardiac fibrosis and promoted the development of capillaries and SMC containing vessels after MI. This combinational therapy was more effective than either treatment individually.

### Hh signaling and cell reprogramming

Reprogramming of somatic cells into cardiomyocytes is another strategy for regeneration of injured heart. Previous studies identified adult cardiac fibroblasts as the most abundant cell population in the heart and as a promising cell source for reprogramming. However, recently Pinto et al. [36] demonstrated that the most prominent heart cells are endothelial cells (around 60%) but not fibroblasts (under 20%). Nevertheless, reprogramming of scar forming fibroblasts into beating cardiomyocytes would be a logical step. Indeed, fibroblasts can be directly reprogrammed into induced pluripotent stem cells (iPSCs) or into cardiomyocytes through the forced expression of pluripotency genes octamer-binding transcription factor 4 (OCT4), sex determining region Y box 2 (SOX2), Kruppel-Like factor 4 (KLF4) and V-Myc avian myelocytomatosis viral oncogene homolog (MYC) or GATA binding protein (GATA4), myocyte enhancer factor 2C (Mef2c), and T-box protein 5 (Tbx5), respectively [37, 38]. iPSCs also have the ability to differentiate into cardiomyocytes [39, 40]. Currently, nothing is known about the role of Hh signaling in fibroblast reprogramming into cardiomyocyte. However, there are some indications that Hh signaling promotes reprogramming of fibroblasts into other cell types. In combination with OCT4 the activation of Hh signaling could reprogram mouse embryonic and adult fibroblasts into induced pluripotent stem cells (iPSCs) [41]. Moreover, mouse fibroblasts could be directly reprogrammed into midbrain dopaminergic neural progenitors by temporal expression of the pluripotency factors and in the presence of Shh and fibroblast growth factor 8 (FGF8) [42]. A combination of the transcription factors achaete-scute homolog 1 (Ascl1), nuclear receptor related 1 protein (Nurr1) with Shh and FGF8b directly reprogrammed embryonic mouse fibroblasts to induced neuronal cells [43].

The P19 cells, a pluripotent mouse embryonal carcinoma stem cell line, can differentiate in vitro into multiple cell types including cardiomyocytes. In the presence of dimethyl sulfoxide (Me_2_SO), P19 cells aggregate and spontaneously differentiate into beating cardiomyocytes [44]. Activation of Hh signaling via overexpression of Shh and Gli2 in aggregated P19 cells induced formation of cardiomyocytes in the absence of Me_2_SO and led to upregulation of expression of Ptc1, Gli1, Gli2 and cardiac muscle transcription factors such as GATA binding protein 4 (Gata4), myocyte-specific enhancer factor C2 (MEF2C), and Nkx2.5 [45]. Inhibition of Hh signaling via depletion of CAM-related/downregulated by oncogenes (CDO), the activator a Hh signaling, in P19 cells or mouse Cdo(−/−) embryonic stem cells resulted in reduced expression Shh, Gli1, Gata4, Nkx2.5 and MEF2C. Moreover, Cdo deficiency caused a significant reduction in cardiomyocyte differentiation and the formation of contractile colonies [46]. These results suggested that Hh signaling was sufficient to promote cardiomyogenesis and it can be used for cellular reprogramming in vivo.

**Fig. 1:**
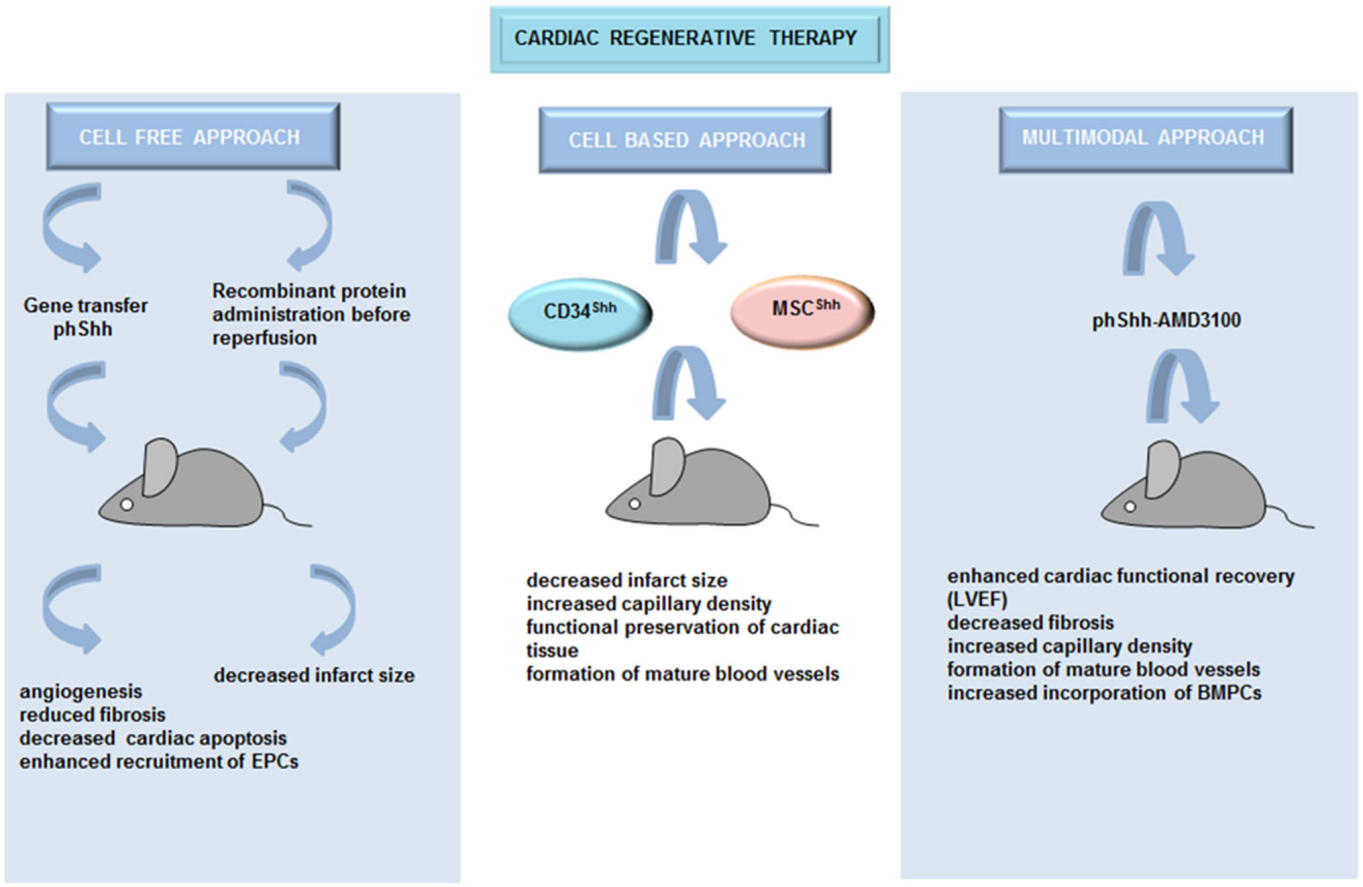
Hh-based cardiac regeneration. Different therapeutic strategies which activated Hh signaling were applied to induce angiogenesis and to target heart regeneration and repair

An overview of the different therapeutic strategies for activating Hh signaling which were applied to target heart regeneration and repair can be found in Fig. 1.

### Future perspectives

There is a strong experimental basis for future studies on the regenerative potential of Hh signaling in the heart. So far, most clinical data have been generated using Hh signaling inhibitors as anticancer therapy in patients with different types of tumors including prostate cancer, basic cell carcinoma (BCC), and medulloblastoma. Increased expression of Hh signaling components is usually observed in tumors. Many small molecules with antagonistic effects on various components of Hh signaling have been identified. Currently, around 90 studies have been either initiated, running or completed with several compounds, which inhibit either Smo or Gli proteins. The use of Hh inhibitors as monotherapy in the clinical setting has been effective for BCC and medulloblastoma. The results of these trials indicate that Hh signaling is an attractive and promising candidate for anticancer therapy.

In case of the ischemic heart, the activation of Hh signaling protects against ischemic injuries. Several therapeutic Hh agonists for ischemia treatment including SAG were also proposed. SAG is a derivative of chlorobenzo thiophene that binds to and activated Smo. It has been shown that up-regulation of the Hh pathway by SAG was efficacious. However, whether SAG can be a proper candidate has to be determined, since it crosses the gut, the placenta and the blood barrier [47–49] and constitutive activation of Hh signaling can result in tumor development. Other Hh agonist drugs with a potential role in tissue regeneration include four fluorinated glucocorticoids halcinonide, fluticasone, clobetasol, and fluocinonide [50], all FDA-approved compounds. These compounds have no apparent association with topical cancers and have been used to treat asthma, inflammation, and skin disease or injury. It has also been shown that fluticasone is well tolerated orally [51]. However, their clinical relevance still has to be evaluated.

Many other questions still have to be answered before Hh based therapy can be applied clinically. What is the molecular mechanism by which exogenous Hh affect in ischemia-induced tissue injury? What component of Hh signaling will be most useful as a target in a clinical setting? Hh proteins are morphogens which have effect on many cell types and cell responses are dependent on the dose of Hh proteins and time of exposure. Can Hh activation increase the potential risk for cancer induction? It is important to determine such factors as right cell type, a right dose, and duration of treatment.

## Concluding remarks

Comparison of the Hh function in regeneration of ischemic heart in lower vertebrates and mammals reveals both similarities and differences. In general, Hh signaling reactivation is a common response to heart ischemia. However, in lower vertebrates, heart ischemia reactivates Hh signaling in epicardial cells and this activation seems essential for cardiomyocyte proliferation, while observations so far in mammals suggest that Hh signaling is crucial for neovascularization.

**Fig. 2:**
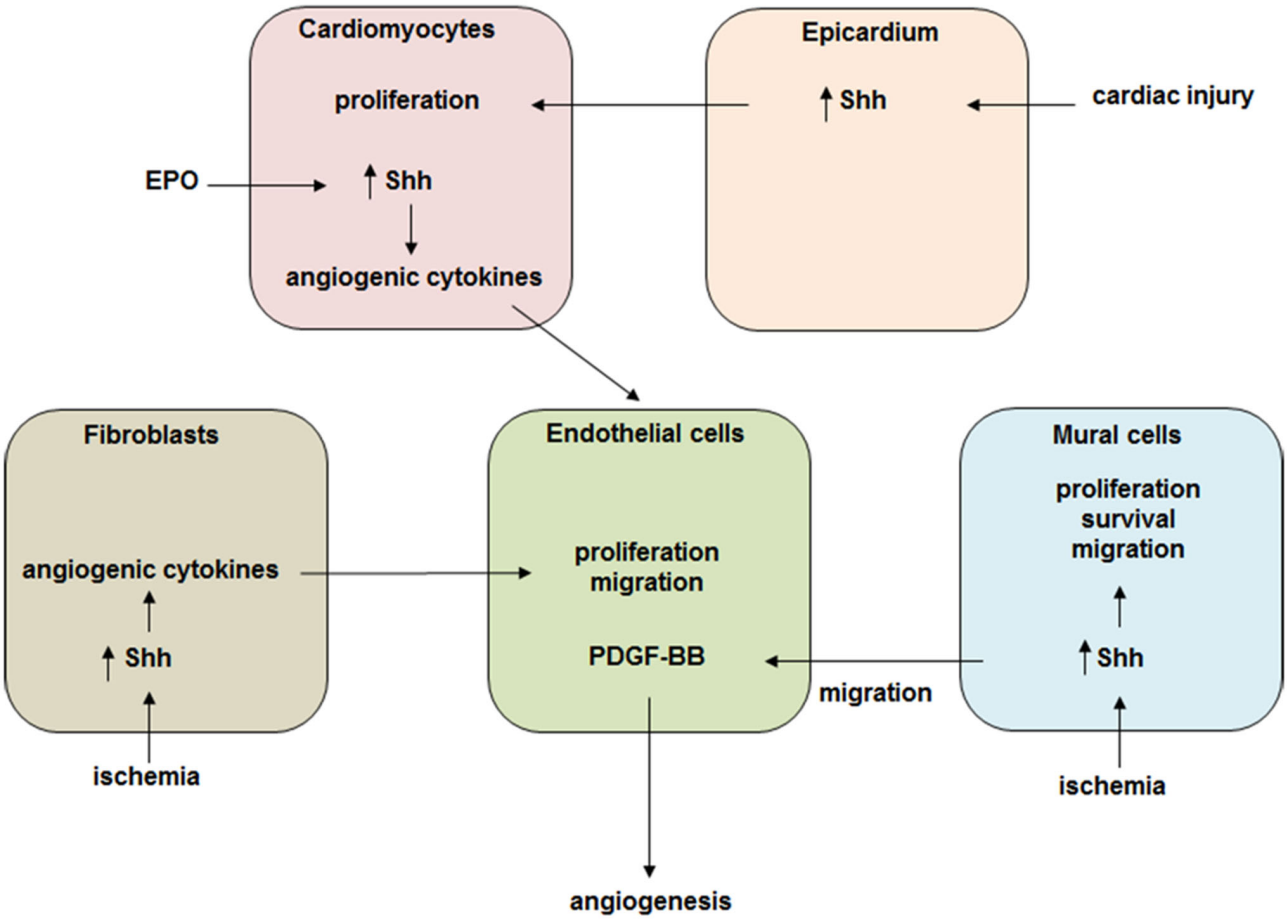
Hh signaling mediates cell–cell cross talk in the ischemic heart. A schematic representation of Hh signaling based cardiac cell interactions. During heart injury it triggers expression of pro-angiogenic cytokines and participates in repair by promoting cell survival, proliferation and migration

Hh signaling has a rather complex effect on the ischemic heart. Its activation in vivo triggers responses in all cardiac cell populations including cardiomyoblasts, endothelial cells, smooth muscle cells and epicardial cells. During injury Hh signaling triggers expression of pro-angiogenic factors such as members of VEGF family (VEGF-A, VEGF-B, and VEGF-C), Ang-1, Ang-2, and SDF-1 in cardiac fibroblast. It also participates in repair by promoting cell survival, proliferation and differentiation. Thus, the alteration of Hh signaling activity in one cell population leads to changes in other cells. We, therefore, suggest the following model for Hh-based cardiac cell–cell interaction (Fig. 2): Hh signaling constitutes an important mechanism to limit the extent of damage following myocardial ischemia and MI by the coordination of various factors. Moreover, reductions in Hh signaling worsen cardiac function and increase infarct size following MI, suggesting that endogenous Hh activity may serve as a biomarker predictive of the extent of recovery after MI. Altogether, Hh signaling can be a potential target for stimulating cardiac regeneration but a more systemic approach like multiscale analysis to study its role in the heart will be required.
